# Nanomaterials—Tools, Technology and Methodology of Nanotechnology Based Biomedical Systems for Diagnostics and Therapy

**DOI:** 10.3390/biomedicines3030203

**Published:** 2015-07-20

**Authors:** Christian Schmidt, Joachim Storsberg

**Affiliations:** Fraunhofer-Institute Applied Polymer Research (IAP), Geiselbergstrasse 69, Potsdam D-14476, Germany; E-Mail: christian.schmidt@iap.fraunhofer.de

**Keywords:** biomaterials, medicine, nano medicine, nano sensors, nano drugs, therapy, diagnostic

## Abstract

Nanomedicine helps to fight diseases at the cellular and molecular level by utilizing unique properties of quasi-atomic particles at a size scale ranging from 1 to 100 nm. Nanoparticles are used in therapeutic and diagnostic approaches, referred to as theranostics. The aim of this review is to illustrate the application of general principles of nanotechnology to select examples of life sciences, molecular medicine and bio-assays. Critical aspects relating to those examples are discussed.

## Significance

Nanotechnology can be likened as a discipline where natural sciences, such as chemical physics and engineering, and life sciences meet, enrich one another and provide a fertile ground for the development of systems with clinical relevance, to name one fast growing area of commercially lucrative research and development. Such development of safe next-generation tools undeniably relies on a robust understanding of nanomaterials.

One area where the detection levels and functionality of nanomedicine is most direly needed is the ability to find a single cancer cell in the context of a multicellular tissue environment without damaging surrounding healthy tissue and also to specifically eradicate this pathological growth with minimal side effects. Great strides have been made toward this goal but many questions remain. Here we use select examples to illustrate how properties of sub-micrometer matter are used in detecting target molecules with great sensitivity, specificity and accuracy.

## 1. Introduction

**Figure 1:**
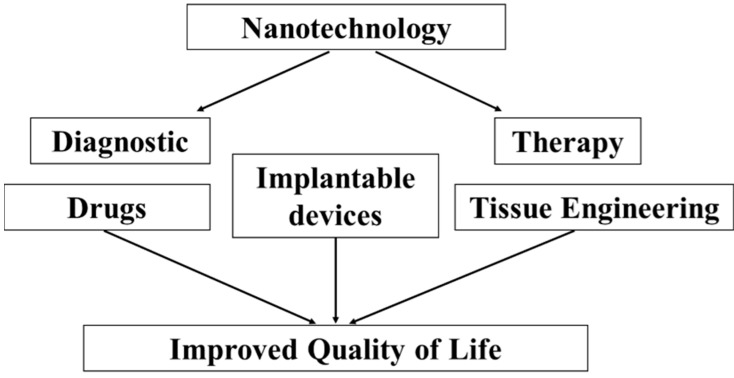
Patients benefit from advances in nanotechnology in medicine. To further our understanding of nanotechnology means to benefit applied research. Applied research in turn then aids the development and production of, e.g., more effective and less costly diagnostic approaches of and treatments for illnesses. Tissue engineering, as another example, also benefits from improvements in nanomedicine with direct benefits for patients, who find quality of life restored and/or improved by more biocompatible implantable devices.

Progress in nanotechnology, which is a multidisciplinary and integrative scientific discipline, improves our daily existence by improving existing systems, processes and materials. The scale of technological advances provided by nanotechnology is illustrated by the emergence of rapidly growing and commercially powerful sub-fields, such as application-oriented nanotechnology and applied nanotechnology with synergistic influences on the progress of basic and customer-oriented medical research. Advances in medical nanotechnology, often referred to as nanomedicine, benefit patients directly by, e.g., improving imaging systems and devices to carry drugs to a targeted location. Cutting-edge developments in these domains now point to a combination of these functionalities to aid diagnostics and therapy (Figure 1).

Research efforts and increased scholarly productivity in various fields of nanomedicine resulted in a steady increase in idea-to-product transfers with considerable commercial success. Predictions of the commercial success range well above and beyond double-digits of billions of Euros in the coming decade with converging forecasts of the most aggressive growth of market opportunities in applied research and commerce in the fields interfacing with function-oriented biomaterial sciences and nano-medicine. This is, however, not restricted to the concept of “enabling functionality” in the sense of drug delivery and generation of new materials; the greatest potential is seen nowadays in areas where products, based on nanotechnology, sometimes ascribed to as nano-products, function by design as smart drugs. Nanomedicine cross-fertilizes the advancement of modern implantable devices by improving our understanding of surfaces and interfaces. Most notably, the success of any implantable device depends on an active management of cellular adhesion, motility and proliferation on its surface. One example is the search for a biocompatible artificial cornea [1,2,3,4,5,6,7,8,9,10,11,12,13,14,15,16,17,18,19,20].

## 2. Definition of Terms Used in This Article

Before we progress in our discussion of nanotechnological applications to life sciences, we will define the terms used in this review.

*Nanotechnology* is defined by us as a scientific discipline aimed at understanding the properties of objects ranging in size from 1 to 100 nm and using the new information for the creation of new processes, devices and materials, including biomaterials, by controlling self-assembly of matter in order to improve mechanical properties and biocompatibility.

We define *drug delivery* as an application and or a development of means to improve pharmacokinetics and directed bioavailability of particles and or molecules for therapeutic and diagnostic purposes. Examples that may come to mind include employment of artificial and bio-compatible nanoparticles or modified peptides; the former class may be based on fullerenes and the latter class could be mimicking bioactive peptides. All these nanoparticles are then transported to the area where carried agents are needed for directed treatment.

In this context, one can easily imagine that a directed and targeted delivery of said agents to the area of need would be mostly beneficial. This, in turn, precludes that the area of need is known. In other words, a means is necessary to correctly define the area of need via appropriate *diagnostics* and subsequent delivery of the needed agent. Ideally, the diagnostic agent should carry the pharmacological treatment, also known as *therapeutic approach*. This combination of *thera*py and diag*nostic* is referred to in this review as *theranostics* [10,11,12,13,14].

## 3. Applications of Nanotechnology to Life Sciences

Applications of nanotechnological knowledge for the improvement and refinement of processes and materials, will now be discussed to illustrate basic concepts used in nanoscience. At first, we wish to direct the attention to a lab on a chip to familiarize the reader with the field of mechanical nano-devices and their applications in screening for active compounds that may inhibit a targeted enzyme, down to a signal to noise ratio where the sensitivity range of single photons can be successfully transduced by micro-cantilevers, effectively connecting micromechanics and single-photon optics. From there, we offer insights into the design of nanoparticles to develop artificial chemical noses and tongues. With this, we guide the reader to the emerging field of designed material to allow for label-free optical detection of biomarkers using a near-quantum-scale effect displayed by nano-sized noble-metal particles and their use in theranostics, biochemical studies of signal transduction in carcinoma cells and studies of metabolism in live model animals. To further explore the area of designed materials, we then briefly discuss biodegradable polymers to aid theranostics in cancer medicine.

### 3.1. Lab on a Chip

The governing principle of nanotechnology is to take advantage of the properties of nanoparticles for the development of devices and processes. The obvious comes to mind: these particles are very small and have a very small mass. This means, however, that additions or subtractions of very small masses should be easily detectable.

One principle that can be used to detect differences in masses uses properties of the harmonic oscillator, where the frequency of the oscillation is indirectly proportional to the mass of the pendulum. The detectable mass difference (Δ*m*) via shifting of the frequency of the harmonic oscillator (Δ*f*) can be approximated to $\frac{\text{Δ}m}{\text{Δf}}\;\approx \;\frac{2m_{osc}}{f_{0}}$ with *m*_osc_ as the oscillator’s mass and its resonant frequency *f*_o_. Recent developments in this field indicate that detection and counting of single molecules appears to be of sufficient precision and accuracy for use in clinical settings (see [21] as a recent review).

**Figure 2:**
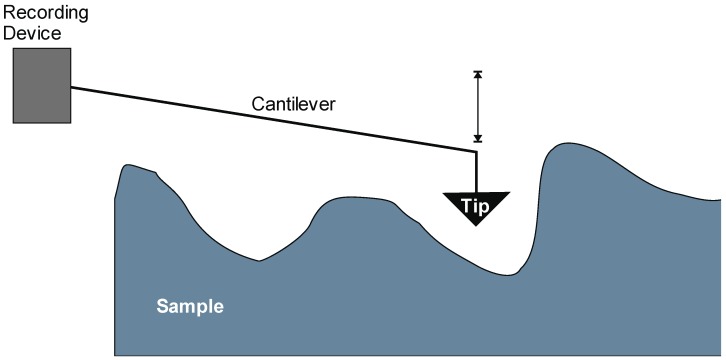
Schematic illustration of the principle of atomic force microscopy. In atomic force microscopy applications, a recording device is used to analyze the movements of a cantilever with a small mass attached to it (see the text for details).

More sensitive than the above-described cantilever is a principle by which adhesion forces between atoms are used to measure forces or distances, as in atomic force microscopy (Figure 2) (AFM) [22]. Here, a very small mass is attached to a cantilever, and the motion of this beam is recorded to measure distances as small as 100 attometers (am; 1 am = 10^−18^ m) [22]. For comparison, the C–H bond has a length of roughly 100 picometers (pm; 1 pm = 10^−12^ m) [23], placing the distance resolution of AFM at two orders of magnitude below the distance of a C–H bond in a biomolecule.

As outlined in the original paper, the sensitivity of AFM critically depends on a low mass of the cantilever, as well as its high deflection for a given force and a resonance frequency greater than 100 Hz to minimize background vibrational noise. Considering that the resonance frequency *f*_o_ is indirectly proportional to the mass of the spring, one is led to the scenario of the limiting practical case that a single atom is used as spring.

In principle, a variety of cantilever-based detection application can be derived from AFM, where a pointed tip is used to trace the surface of a sample. For instance, a ferromagnetic tip could be used to measure electromagnetic forces by deflecting the cantilever. Applications of this could be used in electron transfer studies of chemical reactions. Another application of AFM could be the creation of a bimetallic sensor where differences in thermal expansion coefficients of two materials can be used to measure temperatures by registering cantilever bending. The small mass of the cantilever results in a very small thermal capacity of the sensor, allowing for an almost real-time measurement of thermal events. For example, phase transitions could be monitored using a considerably small amount of sample that needs to be attached to the cantilever. Another scenario could be to employ such a sensor in photo-thermal spectrometric assays. Alternatively, measurements of shifts in resonance frequencies may be used to determine mass changes of a probe attached to the tip of the cantilever, e.g., detection of the gain or loss of mass of a sample due to varying hydratation as a function of temperature to record environmental conditions, such as humidity. By extension, an array of sensors could be assembled to measure an array of parameters, e.g., temperature, humidity and magnetic force, while others serve as reference and internal calibration to insure reproducibility, accuracy and precision.

In this case, a cantilever consisting of a single atom as “sensor” is used to measure forces, such as adhesion, magnetic momenta *etc.* in the sensitivity range of 10 pN, roughly equivalent to the force necessary to rupture an individual hydrogen bond found in a biomolecule [22]. Building on this foundation, Sbaizero *et al.* [23] demonstrated force spectroscopy on a single cell using the well-established fact that the cytoskeleton of a cell is involved in the transduction and transmission of mechanical force [24]. Specifically, Sbaizero *et al.* [23] report that the force needed to remove a covered nanosphere from the surface of a cardiac fibroblast is decreased when the polymerization of the actin filaments is disturbed by pretreating cells with Cytochalasin D. This example illustrates how this method may be used for assessing biophysical properties of the interface between tissue and cells on the one hand and implants or other biomedical devices on the other (see [25] and references therein).

A combination of mechanical and optical nano-devices is the principle of a broad-band all-photonic transduction of nano-cantilevers using a single-mode photonic waveguide. Here, end-coupled and completely transparent nano-cantilevers are separated by a gap of approximately 400 nm. Light passing through the first cantilever then tunnels through the gap and is received via the wave guide of the second nano-cantilever with the resonance frequency being indirectly proportional to the square of the cantilever length, in analogy to the theory of gradient forces used to advance marker detection [26].

An inherent problem of high-intensity signal input-operating devices, such as the above-described approach, is that probabilities of quasi-relativistic couplings between fluctuations in the cavity photon-field and the mechanic oscillator (the cantilever) increase proportionally with the mean photon density in the cavity, resulting in a Gaussian-distributed steady state phenomena due to inherent thermal and vacuum noises. Thus, there is only a limited amount of defined “states” available for proper signal transmission. Refinements of the opto-mechanical near-quantum coupling would mean that, for example, single photons would have to be used for the transmission of signals or the entire input signal needs to be non-linearized, which could be achieved by couplings involving membrane and cavity modes of particles trapped in the cavity for a finite time (see ref [27] for guidance).

Leaving the above-discussed aside, one can formulate the normalized mode functions of the input and output waveguides (abbreviated as *φ*_i_ and *φ*_o_) in a three-dimensional Cartesian system of the dimensions *x*, *y* and *z*. The resulting transmission T from one cantilever to the other is then expressed by T = T (*x*, *y*, *z*). Any out-of plane movement of the cantilevers (*z* + Δ*z*) in turn alters ΔT = T (*x*, *y*, *z* + Δ*z*), such that the linear displacement response *R_O_* as a function of Δ*z* of the output cantilever can be calculated as the ratio of changes of T as a function of the displacement Δ*z*: $R_{o}=\;\frac{\partial }{\partial z}\;\left|{\left[\int_{-\infty }^{\infty }\varphi_{i}\left(x,\;y,\;z\right)\varphi_{o}\left(x,\;y,\;z\;+\;\text{Δ}z\right)dxdy\right]}^{2}\right|$ [25,26,27].

**Figure 3:**
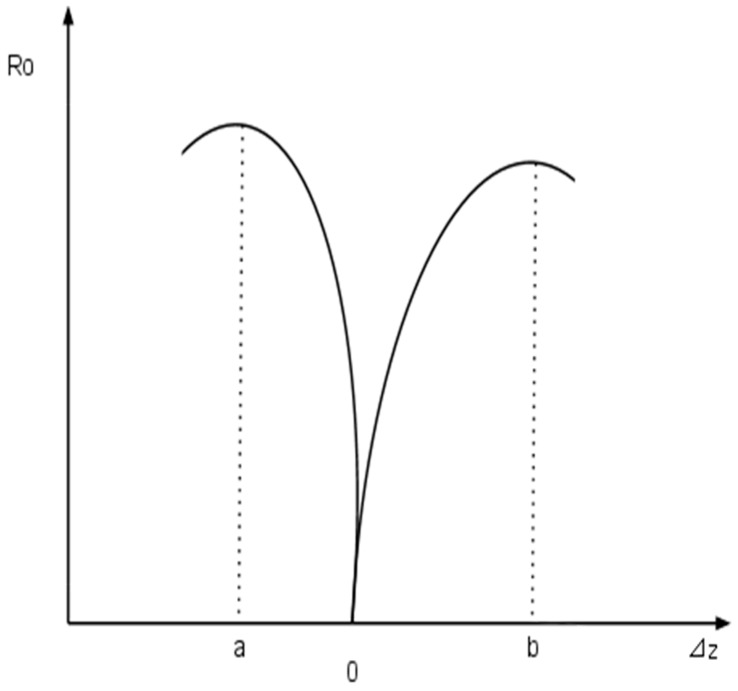
Linear displacement response of cantilevers in broad-band all-photonic transduction using a uni-modular photonic waveguide. Shown here is one graph of possible linear replacement functions *R_O_* for two cantilevers (a; b) as function of the linear replacement Δ*z*, provided that *R_O_* = 0 (internal normalization). Each function for a given cantilever is shown to maximize at different values of Δ*z*, indicating the proper relative position of each cantilever to another in a Cartesian space for optimal transduction efficiency (see the text for details).

Because both cantilevers can function as receiver for a light signal, the optimal linear displacement for the two cantilevers in this apparatus relative to the point Δ*z* = 0 and *R_O_* = 0 needs to be determined. As shown in Figure 3, *R_O_* functions of cantilevers (here named “a” and “b”) do not need to be congruent and the optimal linear out-of plane replacement in relation to the point Δ*z* = 0 (no displacement) has to be determined for the cantilever to safeguard proper functionality (indicated by the points a and b on the abscissa), especially significant for a system, where more than one cantilever is used in signal transmission and processing [25,26,27].

**Figure 4:**
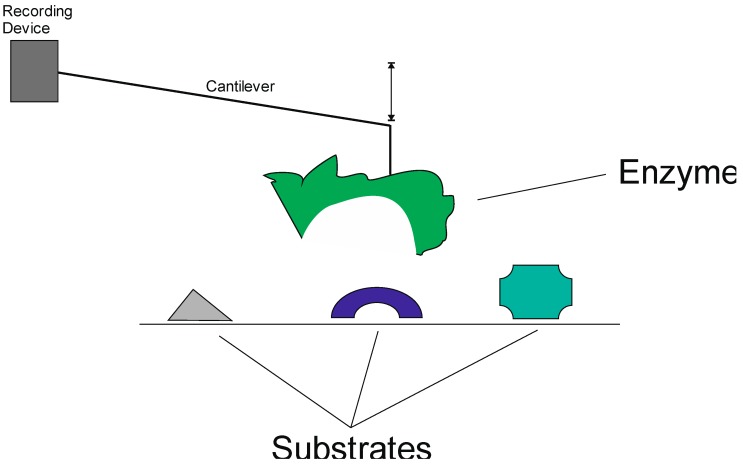
Single-molecule force spectroscopy is an AFM application. An enzyme, attached to a cantilever, is used as sensor, in strict analogy to AFM. As shown here, the enzyme is exposed to an array of substrates, depicted using shapes. The strength of the enzyme-substrate, derived from computer-assisted analysis of the recorded translocation of the cantilever, can then be used to develop biochemical assays (see the text for details).

Another direct application of the AFM principle for the search for bio-active compounds is the single-molecule force spectroscopy (Figure 4) [28]; the authors elected to screen for new inhibitors of the 2-*C*-methyl-d-erythritol-4-phosphate (MEP) pathway designed to specifically affect members of the kingdom bacteria, including human pathogens, such as malaria parasites, but not members of the kingdom animalia (see refs. [29,30], and below for details).

**Figure 5:**
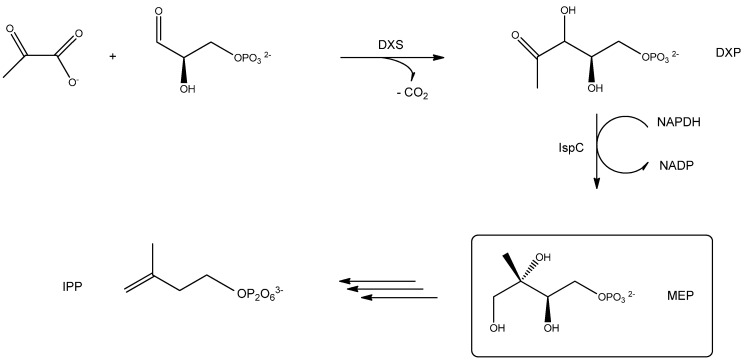
Select steps of the biogeneration of IPP via the mevalonate route in most archaea, fungi and animalia. Three molecules of acetyl-coenzyme A (Ac-CoA) are condensed to hydroxy-3-methylglutaryl-coenzyme A (HMG-CoA) in an HMG-CoA-synthase-catalyzed reaction with a concomitant release of two reduced CoA (HSCoA) molecules. The following endothermic reduction is catalyzed by the HMG-CoA-reductase. Here, reduced CoA is released and Mevalonate is generated, which then, serves as precursor in the biosynthesis of IPP (see text for details).

The MEP pathway (Figure 5) in most bacteria, fungi and plantae is initiated with the condensation of pyruvate with glyceraldehyde3-phosphate, which yields 1-deoxy-d-xylulose-5-phosphate (abbreviated as DXP) and carbon dioxide under the influence of the 1-deoxy-d-xylulose-5-phosphate synthase (abbreviated as DXS). It seems likely that DXS critically depends on Mg^2+^ or Mn^2+^ for its activity and thiamine di-phosphate (TDP) in a reaction mechanism in which pyruvate binds to TDP, followed by a release of carbon dioxide, binding of GAP to the intermediate followed by the terminal release of DXP and TDP to renew the catalytic cycle. The second cascade of reactions involves the reduction and rearrangement of DXP via the 1-deoxy-d-xylulose-5-phosphate reducto-isomerase (abbreviated as IspC) using energy provided by the oxidation of NADPH to NADP, leading to the release of MEP, an intermediate in the biosynthetic path to generate iso-pentenyl-diphosphate (abbreviated as IPP) to provide a basis for downstream biosynthesis of ubiquinone and related compounds [29,30].

**Figure 6:**
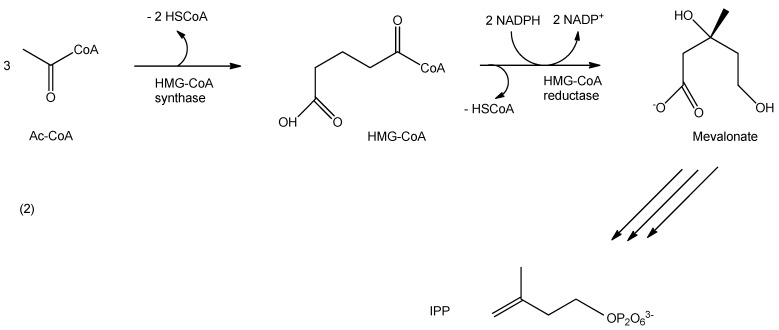
Select steps of the IPP biosynthesis in most bacteria, fungi and plantae. Pyruvate is condensed with glyceraldehyde-3-phosphate to generate 1-deoxy-d-xylulose-5-phosphate (DXP) in the presence of the 1-deoxy-d-xylulose-5-phosphate synthase (DXS), which is then transformed into 2-*C*-methyl-d-erythriol-4-phosphate (MEP) in the presence of the 2-*C*-methyl-d-erythriol-4-phosphate reducto-isomerase (IspC) using energy provided by the hydrolysis of nicotinamide adenine dinucleotide phosphate (HADPH) to nicotinamide adenine dinucleotide (NADH). MEP is then used for further functionalization to yield iso-pentenyl-diphosphate (IPP) as precursor for ubiquinone and its derivatives (see the text for details).

Most archaea, fungi and animalia derive IPP from a different biosynthesis route, the mevalonate pathway (Figure 6).

Here, three molecules of acetyl-coenzyme A (abbreviated as Ac-CoA) are used to form 3-hydroxy-3-methylglutaryl-coenzyme A (HMG-CoA) in the presence of HMG-CoA synthase and the release of two reduced forms of CoA. The HMG-CoA reductase then catalyzes the generation of mevalonate, which now serves as the source for the biogeneration of IPP and subsequent downstream synthesis products [29,30].

As it is clear from Figure 5 and Figure 6, any bioactive compound that is designed to effectively halt synthesis of IPP progenitors in human pathogens, such as malaria parasites should not interfere with the maintenance of the host organism. Therefore, targeted inhibitions of the malarial DXS compartment may serve as potent remedies for malarial infections [28,29,30].

In an attempt to screen for effective DXS inhibitors, Sisquella *et al.* [28] developed a single-molecule force spectroscopy application. Here, an enzyme is C-terminally linked to an appropriately functionalized cantilever instead of the single atom used in AFM techniques. Functionality of the linked enzyme appeared to be unaltered as well as the enzyme’s ability to bind to and discriminate between various substrates in competitive enzyme assays at a single-molecule level. In this first quantitative report, Sisquella and colleagues provided a powerful application of nanotechnology to the field of drug-discovery by improving the sensitivity and accuracy of screening assays by two orders of magnitudes [28]. Recent advantages in the development and refinement of nanodiagnostic platforms for drug discovery are summarized elsewhere, and we urge interested readers to use recent papers and the references therein as a guide to in-depth coverage of the broader field [31,32].

### 3.2. Chemical Noses and Tongues Based on Nano-Biomaterials

Another application of nanotechnology to biosciences is the use of coated nanoparticles for the measurement of concentrations of intracellular ions. For instance, classical methods of measuring [Ca^2+^]_i_ rely on the determination of two rations of fluorescent indicators after a target cell was “loaded” appropriately [33]. This is only valid as long as other cellular content, such as proteins, do not interfere with the dye-Ca^2+^-interaction. Published evidence supports the notion that non-calcium interactions with commonly used dyes need to be taken into account [34,35]. This has driven a considerable move to improve Ca^2+^ indicators. A recent report, for example, strongly argues for the use of a dye, based on a green fluorescent protein, which was originally isolated from the jelly fish *Aequorea victoria* [36,37]. The authors concede that this improved indicator is susceptible to pH changes and that a correction of pH effects is necessary for qualified statements based on the use of their dye [34], which is in accord with published evidence [38,39,40]. In addition to the issues raised, there is always the problem of local distribution of dyes and their references.

**Figure 7:**
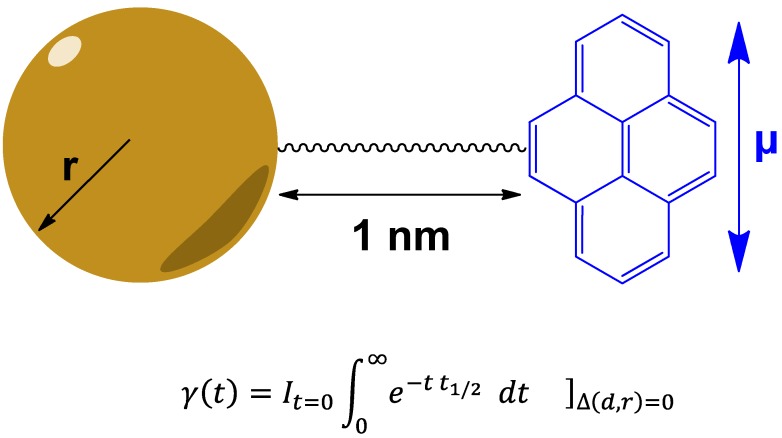
Small gold particles quench the fluorescence intensity of a nearby chromophore. A small gold particle with the radius r can quench the fluorescence intensity of a chromophore with the magnetic moment *µ* oriented perpendicular to the nanoparticle-chromophore axis of 1 nm according an exponential decay *γ* (expressed as function of time with *r* and *d* being held constant (see text for details).

The recent publication of functionalized nanoparticles, or “chemical noses” [41], appears to be an indication of the way forward by taking advantage of the size of nanoparticles and chemical functionalization to allow local determination of concentration of biomolecules. In this case, the authors used the ability of a small gold particle of 4 nm diameter to quench the intensity I of a given fluorescence at time *t* = 0, with the half-life *t*_1/2_, from a constant distance *d* from the chromophore to the nanoparticle and with the dipolar moment of the chromophore *µ* oriented perpendicular to the nanoparticle-chromophore-axis (Figure 7), according to the equation $I\int_{0}^{\infty }e^{-t\;t_{1/2}}dt$ [42].

Given that the quench efficiency is indirectly and monotonically proportional to the particle size, average fluorescence lifetimes range from around 1.7 × 10^−10^ s (170 ps) for an Au nanoparticle of a 30 nm radius, to ~100 ps for a 15 nm particle and 72 ps for 1 nm radii, given a constant distance of the fluorochrome to the gold particle of 1 nm, amounting to a twenty-fold decreased fluorescence time of a dye conjugated at a distance of 1 nm to a gold particle of a radius on 1 nm in comparison to the free dye’s fluorescence lifetime of ~1.5 ns (1.5 × 10^−9^ s).

Yu and coworkers [41] used gold particles with a radius of approximately 2 nm that were functionalized with a hydrophobic core to insure stability of the device, followed by a layer of poly-(ethylene glycol) designed to optimize biocompatibility and surface-charged residues to interact with target proteins. Successful interaction with target proteins then released fluorescent dye, which was then used as a read-out. The inherent properties of the system was designed to produce a very short-lived fluorescence signal in the case of unbound protein, whereas bound protein lead to a release of a considerably long-lasting fluorescence signal. Using classical multi-color detection approaches in combination with this nanotechnological application, the authors could distinguish targeted proteins, which normally display absorbance maxima at 280 nm incident light [41]. A recently published review illustrates that progress has been made in the detection of vaporized molecules in human breath as a means to sample biomarker for diagnostic purposes (see [43] and references therein).

### 3.3. Theranostics Based on Quasi-Relativistic Effects

**Figure 8:**
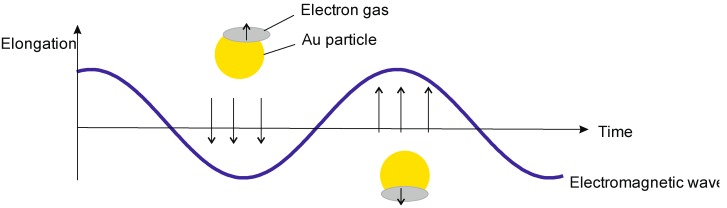
Surface plasmon waves can be regarded as oscillations of the electron gas of a noble metal nanoparticle in response to irradiation with polychromatic light. As shown here, the electron gas of a gold particle of the size below the wavelength of the irradiating light responds to the dislocation in response to the electromagnetic wave with an oscillation (see text for details).

Nano-spheres of noble metals, free of any coating, display one additional property, seen at the interface of two media with a real and an imaginary refractive index. Irradiation with polychromatic light leads to an oscillation of charge density relative to the framework of nuclei and based on Coulomb attraction manifests itself as a transverse-magnetic polarized wave (named surface plasmon wave (SPW, Figure 8)) with its maximum at the interface and decay in both media. The SPW magnetic vector is localized at a right angle to the SPW propagation vector and plane parallel to the interface with its propagation constant β described as $k{\left(\frac{en^{2}}{e\;+\;n^{2}}\right)}^{\frac{1}{2}}$, with *k* as the free space wave number, *e* as the dielectric constant of the metal and n as the refractive index of the dielelectric material [44]. As for nanoparticles and nano-spheres, they scatter white light as a result of SPW and emit light, similarly to fluorescent probes. This oscillation frequency of the resulting SPW depends on density and distribution of the conducting electron plasma [44,45,46].

One additional property of these nano-sized noble-metal spheres needs to be considered: Couplings of such spheres, in strict analogy to the concept of orbital hybridization between single atoms, leads to new properties of such grouped objects with a significant sensitivity to deformation and vibration. Expressed mathematically for a simple two-center system with a distance d between the coupled particles, changes of the plasmon frequence shift (Δλ) are inversely proportional to the ratio of distance between resonating bodies and the length of the entire object *l*, with τ given as the decay constant of the plasmon signal and β being the maximal fractional plasmon resonance wavelength shift: $-\tau {\text{ log}}_{\beta }\text{Δλ}=\sqrt[{3}]{\frac{d}{l}}$ [47].

The log-scale and indirect proportionality between Δλ on the one hand and *d* and *l* on the other provides a basis for the utilization of coupled nano-rod arrays with broken symmetry for photovoltaic applications due to uniformly constructive interference over a broad range of incident light wavelengths resulting in a uni-directional broadband output in the case of honeycomb arrays to the development of beam-steering devices for optical photonic-ciruit supported signal processing based on a phased array of stacked 3-D optical Yagi-Uda nano-antennae (see references [48,49] for details).

Another direct application of quantum-dot optical coupling in life sciences and oncology is the detection of cancerous lesions using anti-epidermal growth factor receptor type II antibody (abbreviated as α-EGFRII) coated noble-metal nano-shells [50,51,52]. In its essence, this nano-technological approach relies on the utilization of knowledge that a mutated form of the product of the oncogene *new*, where amino acids 6 to 273 in the extracellular domain are lost and a glycine is created at the new fusion point, functions as a tumor-specific decoration of the cell surface; antibodies directed against this mutant are currently being used in oncology research (see references [53,54,55,56,57,58] for further reading). In addition to its already known functions, levels of circulating EGFRII are indicative of multi-treatment resistance of cancers (reviewed in [59]). Thus, a directed targeting of cancerous cells to deliver tailored treatment could be an approach to overcome multi-drug resistance of this devastating disease (see [60,61] for further reading).

### 3.4. Cancer Nanotechnology

Early recognition of cancerous lesions depends on a complete understanding of the collective features of genesis and progression of tumors. Such knowledge would, in turn, allow a targeted delivery of drugs. Here, the combination of diagnostics and therapeutics, in the form of nanomaterial-derived theranostics, seems to be a promising avenue to precisely target malignancies at the cellular level and deliver tailored treatments to the appropriate recipient, thus, reducing patients’ burden caused by high-dosage application of pharmaceutical remedies. All these approaches, however, hinge on a full understanding of all aspects of cancer biology, such as the biology of oncogenes and tumor-suppressors and the role of the tumor environment in the genesis and the progression of cancers, as well as the influence of hypoxia exerted on the properties of cancers (see [62,63,64] and references therein as guides to principles and recent advantages).

From a standpoint of translational potential in the sense of immediate relevance to the applied nanotechnology branch, with focus on the pursuit of commercially viable avenues relevant and attractive to clinicians and small and medium enterprises alike, a few randomly chosen examples may serve as cases in point.

For instance, Chen *et al.* [65] synthesized dually, pH and temperature sensitive micelles, tailored to the tumor environment via composition of the co-polymer used (see [66,67,68] as a backdrop). The dually sensitive di-block-co-polymer with Poly(*N*-(2-hydroxypropyl) methacrylamide diacetate) being a thermosensitive material suitable for the intended delivery of the payload, paclitaxel; see [66,67] for background on thermo-responsive polymers. Whilst the authors see a slight albeit significant increase in tumor size in the mouse model used, no significant loss of body weight of the animals was observed. Of note is that no significant levels of loaded carriers were found in the liver, although no evidence was presented to address the issue of particle clearance [65].

Another area in the field of cancer nanotechnology cannot be neglected: the mode of drug delivery. For instance, Parcado *et al.* [69] review progress made on refinement of programmable nanomedicine, namely synergistic and sequential drug delivery systems. The former is designed to deliver payload upon recognition of two or more stimuli, whereas the latter is designed to release payload once individual stimuli are recognized. Both are not mutually exclusive from one another and represent promising platforms for the refinement of potential therapeutic approaches. Further effort is required before a translation into application-relevant prototypes of pharmaceutic formulations can be considered feasible. One area is the optimization of the pharmacokinetic characteristics to secure spatial-temporal precision of the recognition of the stimulus required for release of the payload. Another issue that needs addressing is the simplification of the structures required. This will most likely improve commercial viability by simplification of the approval process if, say, existing delivery systems can be combined to achieve a programmable drug delivery system [69].

Another promising avenue of cancer nanotechnology lies in the generation of delivery systems that form suitable carriers in the bottom-up strategy of assembly (see [70,71,72,73] and references therein). Wei *et al.* [74] used the feature of amphiphilic dendrimers [75] to form nanomicelles [76]. Using immunocompromised NSG mice (see [77,78] for mouse strain description), the authors found that tumor volume was reduced after treatment with Dox-loaded carriers while the mice retained body weight [74]. Whether the formulation generated by Wei *et al.* [74] could be reasonably compared with other formulations, such as the ones reported in [79,80], with regard to efficacy and safety or mechanism of action is yet to be determined.

### 3.5. Chemically Functionalized Quasi-Atoms in Cancer Theranostics

**Figure 9:**
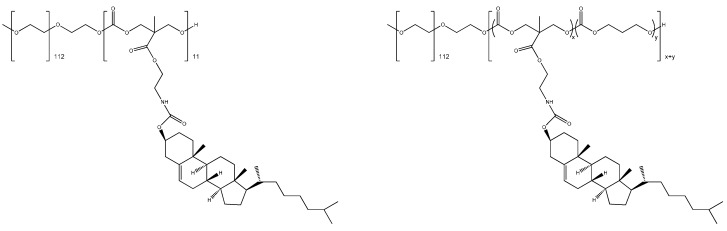
Structures of some biodegradable and amphophilic co-polymers with Cholesterol as functionalization to target tumor cells. See the text and Lee *et al.* [88] for details.

Lee *et al.* [81] designed, created and tested biodegradable amphiphilic nanoparticles using cholesterol as functionalization (Figure 9) to preferentially target tumors in inoculated nude mice with minimal toxicity for normal cells and the host organism. By extension, usage of relativistic effects, such as forces generated by permutations of gradient fields and scattering photons, could be used to deliver a nano-micro syringe with a nanoparticle for individual treatment of cells [82].

Another hallmark of cancers is a constitutively increased cellular pH compared to the adjacent extracellular space with implications for alterations in oncogenesis and malignant behavior in the creation and maintenance of appropriate micro-environments for cancer stem cells [83,84,85,86,87]. In its essence, the presence of cancer stem cell niches with an alkaline pH environment could favor the survival of tumor stem cells by providing a climate in which those cells can successfully resist conventional drug treatment. If so, a targeted delivery of anti-cancer drugs at the precise pH and temperature of the tumor cell niche could represent a reasonable approach for treatments of multi-drug resistant cancers. Pioneering efforts by Kim *et al.* [88] using new compounds resulted in a controlled release of drugs at shifted pH at 40 °C with Böhm *et al.* [89] reporting temperature and pH-controlled release of loaded agents at room temperature, opening the field to the generation of future therapeutic approaches, see [90,91] for recent examples.

## 4. Comparing Theranostic Features Using a Better than a Random Guess Value

**Figure 10:**
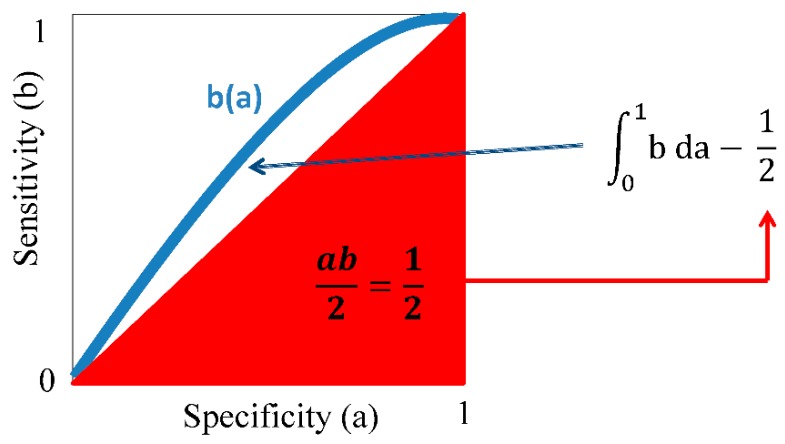
Derivation of a factor better than a random guess from the area under the standardized operating characteristics curve. Shown here are specificity (a) and sensitivity (b) normalized to 1 and the area under the standardized operating characteristics curve (AUC). If a = b can be used to describe a random guess, subtracting the area of the right triangle (in red) from AUC, a factor is obtained that, when multiplied with 100, shows how much better an assay is than the random guess (see text for details).

To ease comparisons of diagnostic approaches, determination of the area under the receiver operating characteristic curve (AUC, defined by $\int_{0}^{1}\text{b da}$) is more and more reported in life sciences publications (see [92,93] for examples). Simple geometry can be used to derive a term *b*_rag_ for better than a random guess by subtracting 0.5 from AUC, taking into account the area of the right triangle located underneath the function b (Figure 10). Factoring in 100 into *b*_rag_ now yields a percent value that is above the random guess curve, defined by the term b (a) = a.

Using this new “*b*_rag_” value, we could roughly compare the above the random guess quality of attempts to detect early manifestations of breast cancer using tear proteomics (*b*_rag_ = 25%; [92]) to the more invasive collection of serum and determination of a micro-array signature of auto-antibodies (*b*_rag_ = 25.6%; [93]). Both attempts are roughly equally above random guess with the collection of tear fluid being a less invasive harvest of material than the collection of a patient’s serum. This factor may also be used to assess the prognostic value of suggested markers for the detection of tumors. Li *et al.* [94] report serum markers used for the differentiation between tumor stages with accompanied AUC values ranging from 0.4416 (*b*_rag_ = −5.84% (worse than a random guess)) to 0.6234 (*b*_rag_ = 12.34% (better than a random guess)). As with all comparative measures, this factor can only be used if study cohorts and methods, such as statistics and data collection, can truly be compared between AUC values. We therefore urge caution in using this value. As a case in point, the *b*_rag_ values obtained from Li *et al.* [94], see above, cannot be seen in the context of, say, AUC values derived from an in-depth Memorial Sloan Kettering Cancer Center analysis of exclusion-prediction of mammary carcinoma metastases to sentinel lymph node [95]. In comparing predicted probability of metastasis to actually observed probability, the authors found “comparable” probability (AUC = 0.722 (*b*_rag_ = 22.2%; better than a random guess); the quote is taken from the abstract of [95]).

## 5. Perspective

Nanotechnology is, undeniably, an interdisciplinary endeavor. As our understanding of small matter, in this case nanomaterial, improves, so does our ability to translate the underlying principles into paradigms of the generation of tools and methods to better our lives and deepen the intellectual grasp of our surrounding environment. In this article, we illustrated on select examples how properties of nanomaterials, with such small mass and quantum-related effects (e.g., energy transfer or resonance phenomena) can be used to measure and count single molecules with direct consequences on the refinement of, say, cancer theranostics, with regard to detection and counting of biomarkers. At the same time, the tools and methods developed can only be as precise and accurate as the underlying premises, hence, our ability to explicate phenomena. A case in point may be our fragmented comprehension of cancer biology or immunology, to name two areas of life sciences. Sidney Brenner’s phrase “(…) we are drowning in a sea of data and starving for knowledge (…)” [96] elegantly describes the so-far not adequately addressed issue of extracting knowledge from the existing body of published work. With the advent of the relative young discipline of nanotechnology, nanoinformatics, as referred to in [97,98,99], is positioned to aid in filling in this void. With so much achieved already, we are confident that nanotechnology will attract many talented researchers and groups of labs to further explore nature’s gift to nanoscience.

## Other Remarks

Space considerations forced us to select citations and limit the scope of this review. Our selection of citations does not imply that we believe the papers cited are superior to the references available for citation. We, therefore, wholeheartedly apologize to all colleagues whose significant contributions are not cited in this paper.

## Abbreviations

Ac-CoA: acetyl-coenzyme A
AFM: atomic force microscopy
AUC: area under the receiver operating characteristic curve
*b*_rag_: better than a random guess
α-EGFRII: anti-epidermal growth factor receptor type II antibody
DXP: 1-deoxy-d-xylulose-5-phosphate
DXS: 1-deoxy-d-xylulose-5-phosphate synthase
EGF: epidermal growth factor
EGFRII: epidermal growth factor receptor type II
GAP: glyceraldehyde 3-phosphate
HMG-CoA: hydroxy-3-methylglutaryl-coenzyme A
IPP: iso-pentenyl-diphosphate
IspC: 1-deoxy-d-xylulose-5-phosphate reducto-isomerase
MEP: 2-*C*-methyl-d-erythritol-4-phosphate
NADH: nicotinamide adenine dinucleotide
NADPH: nicotinamide adenine dinucleotide phosphate
SFM: single-molecule force spectroscopy
TDP: thiamine di-phosphate
